# Cationic Porphyrin Induced a Telomeric DNA to G-Quadruplex Form in Water

**DOI:** 10.1155/2008/294756

**Published:** 2008-03-02

**Authors:** Takashi Murashima, Daichi Sakiyama, Daisuke Miyoshi, Masanori Kuriyama, Takashi Yamada, Toshifumi Miyazawa, Naoki Sugimoto

**Affiliations:** ^1^Frontier Institute for Biomolecular Engineering Research (FIBER), Konan University, 8-9-1 Okamoto, Higashinada-ku, Kobe 658-8501, Japan; ^2^Department of Chemistry, Faculty of Science and Engineering, Konan University, 8-9-1 Okamoto, Higashinada-ku, Kobe 658-8501, Japan

## Abstract

The formation of the DNA G-quadruplex is induced by the addition of a novel porphyrin carrying four cationic tethers. Circular dichroism spectroscopy reveals that the porphyrin binds to *Tetrahymena* telomeric repeat to form G-quadruplex under stabilizing-cation-deficient and no buffer conditions.

## 1. INTRODUCTION

Guanine-rich tandem repeats can be folded to form quadruplex structures through Hoogsteen hydrogen bonding [1]. These guanine-rich segments are found in biologically important regions such as telomeres [2], *c-myc* gene promoter regions [3], immunoglobulin switch regions [4], and fragile X-syndrome triplet repeats [5]. Not only because their biological importance but also because the potential applications in supramolecular chemistry and nanotechnology, a G-quadruplex attracted much attention in the field of medicinal chemistry, pharmaceutical biology, and material sciences. For example, telomere sequences have been used for the construction of nanomolecular machines [6–8] and DNA logic gates [9–11].

Because the G-quadruplex polymorphism and the duplex-quadruplex conversion are affected by environmental factors, we [12–14] and others [15, 16] have investigated the structure and thermodynamic stability of DNAs under molecular crowding conditions as a mimic of the cellular conditions. Another important factor for the stabilization and polymorphism of the G-quadruplex structure is the presence of certain metal cations, effectively K^+^ and Na^+^ [1, 17, 18]. Most of the previous studies about the structure and stability of G-quadruplex were conducted in cation-containing solutions, and to our knowledge, only a few reports have been published using salt-deficient conditions in buffer solution [19–28]. Since DNA secondary structures such as quadruplex exist only within a narrow range of biophysical conditions or their mimic conditions, chemical probe that traps quadruplex structure under simple conditions is desired. Here, we report that small organic molecule induced a telomeric DNA to G-quadruplex formation under noncrowding, and no buffer conditions in the absence of added cation.

## 2. EXPERIMENTAL

### 2.1. Materials and methods

Most of the reagents and solvents were purchased from Wako Pure Chemical Industries, Ltd., Tokyo Kasei Kogyo Co., Japan, and Sigma-Aldrich Co., Mo, USA, and used without further purification. ^1^H NMR spectra were recorded on a Varian UNITY 300 spectrometer at 299.94 MHz by using CDCl_3_ or CD_3_OD as a solvent and tetramethylsilane as an internal standard, and *J* values are given in Hz. Mass spectra were measured with a PerSeptive Biosystems Voyager DE-Pro. Circular dichroism (CD) spectra of DNA quadruplexes were obtained by using a Jasco J-820 spectropolarimeter.

### 2.2. Synthesis of a novel cationic porphyrin

**Scheme 1:**
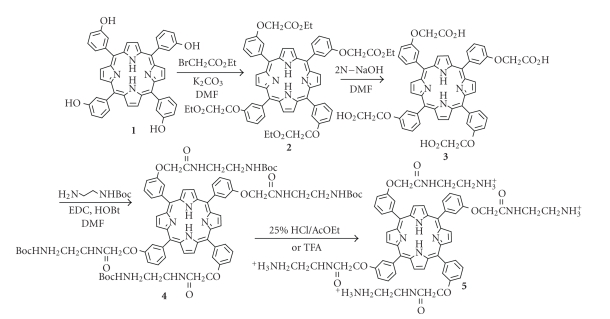
Synthesis of cation tethered porphyrin **5**.

As a G-quadruplex-inducing agent, a novel cationic porphyrin **5** was prepared according to Scheme 1. The molecule **5** was designed to bind to G-quadruplex by a stacking interaction between porphyrin ring and G-quartet plane, together with electrostatic interaction between ammonium cations and DNA backbone. In spite of a postulated low affinity for G-quadruplex that is due to the bulky substituents, porphyrin **5** is expected to grab at the G-quadruplex with the corporation of *π*-stacking and electrostatic interactions. All the reactions were conducted in the dark.

### 2.3. Preparation of 5,10,15,20-tetrakis(3′-hydroxyphenyl)porphyrin 1

To a solution of 3-hydroxybenzaldehyde (3.66 g, 30 mmol) in propionic acid (70 mL), was added pyrrole (2.40 g, 36 mmol) at 110°C under nitrogen atmosphere and stirred for 1 hour. Then, the solution was stirred over night at room temperature under air to afford oxidation of the precursor to porphyrin. The solvent was removed and the residue was purified by silica gel column chromatography using chloroform/methanol = 7/1 as an eluent to obtain compound **1** as a purple crystal (1.06 g, 21% yield).

### 2.4. Alkylation of phenolic hydroxyl groups of 1

To a solution of **1** (0.34 g, 0.5 mmol) in DMF (10 mL) were added potassium carbonate (5.5 g, 40 mmol) and ethyl α -bromoacetate (3.4 g, 20 mmol). The mixture was stirred at room temperature for 24 hours. The reaction was quenched with water (100 mL) and the mixture was extracted with chloroform (3 × 30 mL). The combined organic phase was washed with water (3 × 30 mL) and brine (1 × 30 mL), then dried over Na_2_SO_4_. The solvent was removed and the residue was purified by silica gel column chromatography using chloroform/ethyl acetate = 10/1 mixed solvent as an eluent to give compound **2** as a purple crystal (0.42 g, 82% yield); ^1^H NMR (CDCl_3_, 300 MHz) δ-2.84 (s, 2H, NH), 1.25 (t, 12H, *J* = 7.2, CH_3_), 4.26 (q, 8H, *J* = 7.2, CH_2_CH_3_), 4.80 (s, 8H, OCH_2_), 7.34 (dd, 4H, *J* = 2.9, 8.6, Ar-H), 7.64 (t, 4H, *J* = 8.1, Ar-H), 7.75 (s, 4H, Ar-H), 7.83 (d, 4H, *J* = 7.2, Ar-H), 8.84 (s, 8H, pyrrole-*β*-H); *m/z* (MALDI-TOF) 1023 (M^+^ + H, 100%).

### 2.5. Hydroxylation of 2

To a solution of **2** (0.31 g, 0.3 mmol) in DMF (250 mL) was added 2N NaOHaq (50 mL). The solution was stirred over night at 50°C. The solution was concentrated to a total volume of 150 mL, and the pH of the solution was adjusted to 2.0 with 1N HCl to give a green precipitate. The precipitate was washed with chloroform to give compound **3**. This compound was used for the next reaction without further purification.

### 2.6. Amidation of 3 with monoprotected ethylenediamine

To a solution of **3** (45 mg, 0.05 mmol) in DMF/DMSO = 3/1 (4 mL) were added *N*-*tert*-butoxycarbonylaminoethylamine (40 mg, 0.25 mmol), 1-hydroxybenzotriazole (HOBt) (41 mg, 0.3 mmol), and 1-ethyl-3-(3-dimethylaminopropyl) carbodiimide (EDC) (58 mg, 0.3 mmol). After the mixture was stirred over night at room temperature, the reaction was quenched with water (50 mL). The resulting mixture was extracted with dichloromethane (3 × 50 mL) and the combined organic layer was washed with saturated NaHCO_3_aq (3 × 30 mL) and brine (3 × 30 mL), then dried over Na_2_SO_4_. The solvent was removed and the residue was purified by silica gel column chromatography using dichloromethane/ methanol = 15/1 as an eluent to give compound **4** as a purple crystal (0.059 g, 79% two steps overall yield); ^1^H NMR (CDCl_3_, 300 MHz) δ-2.83 (s, 2H, pyrrole-NH), 1.31 (s, 36H, *tert-Bu*), 3.31 (q, 8H, *J* = 5.3, CH_2_), 3.48 (q, 8H, *J* = 5.6, CH_2_), 4.69 (s, 8H, OCH_2_), 4.93 (brs, 4H, NH), 7.34 (dd, 4H, *J* = 2.1, 8.1, Ar-H), 7.40 (brs, 4H, NH), 7.65 (t, 4H, *J* = 8.1, Ar-H), 7.81 (s, 4H, Ar-H), 7.87 (d, 4H, *J* = 7.8, Ar-H), 8.83 (s, 8H, pyrrole-*β*-H).

### 2.7. Preparation of cationic porphyrin 5

A solution of **4** (29 mg, 0.02 mmol) in trifluoroacetic acid (5 mL) was stirred at room temperature for 2 hours. After the solvent was evaporated off, diethyl ether (50 mL) was added to give a green precipitate. To the solution of the precipitate in water was added excess NaOHaq. and the resulting solid was dissolved in chloroform. 1 M HCl was added to this solution to give green precipitate of porphyrin tetra hydrochloric acid salt **5** (22 mg, 92% yield); ^1^H NMR (CD_3_OD, 300 MHz) δ 3.20 (t, 8H, *J* = 5.3, CH_2_), 3.70 (t, 8H, *J* = 5.6, CH_2_), 4.94 (s, 8H, OCH_2_), 7.69 (dd, 4H, *J* = 2.6, 8.3, Ar-H), 7.99 (t, 4H, *J* = 8.1, Ar-H), 8.20 (s, 4H, Ar-H), 8.23 (brs, 4H, Ar-H), 8.85 (s, 8H, pyrrole-*β*-H); *m/z* (MALDI-TOF) 1079 (M^+^ + H, 100%).

## 3. RESULTS AND DISCUSSION

*Tetrahymena* telomeric sequence d(T_2_G_4_)_4_ was chosen as a model molecule and the cationic porphyrin **5** was used as a G-quadruplex inducing agent. Besides the effect of monovalent cation, the difference between intrastrand and interstrand G-quadruplexes was also examined to compare sequences d(T_2_G_4_)_4_ and d(TG_4_T_2_G_4_T).

**Figure 1:**
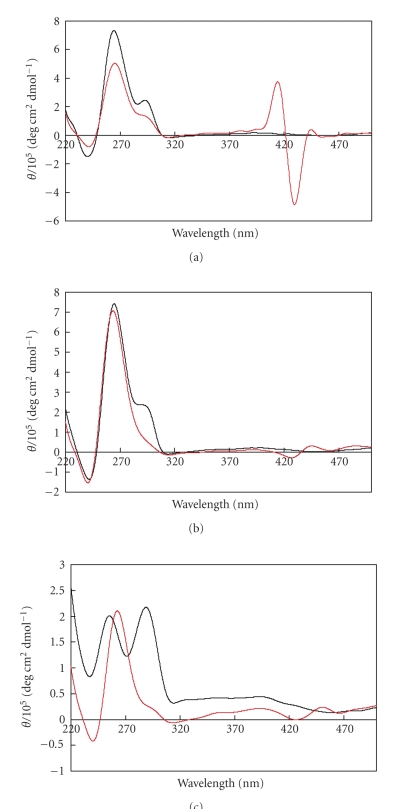
CD spectra of d(T_2_G_4_)_4_ (25 *μ*M) with (red) or without (black) porphyrin **5** (75 *μ*M) in buffer containing 100 mM KCl and 50 mM Tris-HCl (pH 7.5) (a), in 100 mM KCl (b), and in water (c) at 0°C.

The structure of d(T_2_G_4_)_4_ in the presence and absence of cationic porphyrin **5** was verified by circular dichroism spectroscopy in a buffer containing 100 mM KCl and 50 mM Tris-HCl (see Figure 1(a)), in 100 mM KCl solution (see Figure 1(b))), and in water (see Figure 1(c)). CD spectrum of (T_2_G_4_)_4_ without porphyrin **5** had shoulder around 295 nm (see Figures 1(a) and Figure 1(b)) or positive peak around 295 nm (as shown in Figure 1(c)). These CD spectra indicate a hybrid G-quadruplex structure of (T_2_G_4_)_4_ without porphyrin **5**, whose CD spectrum has positive intensities both at 260 and 295 nm. On the other hand, the positive peak or shoulder around 295 nm disappeared by the addition of the porphyrin. This indicates that a structural transition of (T_2_G_4_)_4_ from a hybrid to a parallel G-quadruplex was induced by the porphyrin. Therefore, these results lead us to conclude that porphyrin **5** induces a parallel G-quadruplex structure of (T_2_G_4_)_4_ under various conditions. Bisignate signals of porphyrin were strongly induced in a buffer solution, suggesting that the porphyrin was stacked on the G-quadruplex surface. Relatively, weak signals for the porphyrin were observed in solutions without Tris-HCl. The self-aggregation of porphyrin in such solutions seems to be the reason for these weak interactions.

We investigated the CD spectral changes of d(T_2_G_4_)_4_ by the addition of porphyrin **5** in the absence of added cation (see Figure 1(c)). All DNA samples with or without porphyrin were annealed by heating to 90°C followed by slow cooling to 0°C over 8 hours. Both spectra shown in Figure 1(c) were measured using a d(T_2_G_4_)_4_ solution in Milli-Q water with no addition of external cation. In the absence of cationic porphyrin **5**, d(T_2_G_4_)_4_ did not form a G-quadruplex. However, when the sample was prepared in the presence of porphyrin **5**, the CD spectrum exhibited both a positive signal around 260 nm and a negative signal around 240 nm, indicating the formation of the d(T_2_G_4_)_4_ G-quadruplex. A weak induced CD signal for the porphyrin **5** was also observed between 420 and 460 nm. The induced CD signal is additional evidence for the interaction between d(T_2_G_4_)_4_ and **5**, since its appearance in the CD spectra is indicative of the change in the chirality of the proximal chemical environment of **5**.

**Figure 2:**
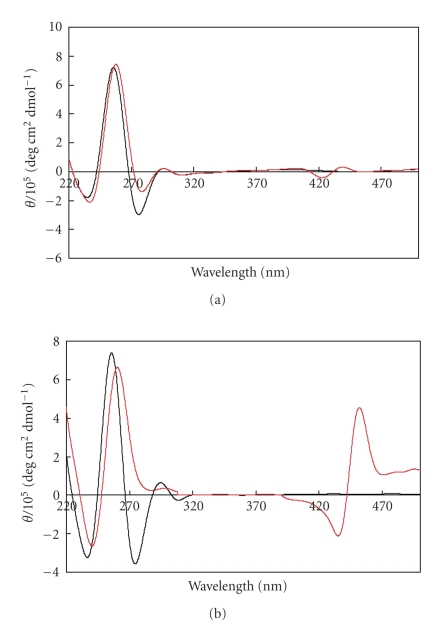
CD spectra of d(TG_4_T_2_G_4_T) (25 *μ*M) with (red) or without (black) porphyrin **5** (75 *μ*M) in buffer containing 100 mM KCl and 50 mM Tris-HCl (pH 7.5) (a) and in water (b) at 0°C.

The structure of a half-length of *Tetrahymena* telomere sequence, d(TG_4_T_2_G_4_T), was studied by CD spectroscopy. CD spectra of 50 *μ*M of d(TG_4_T_2_G_4_T) (G-quadruplex concentration: 25 *μ*M) in a 100 mM KCl solution had positive and negative peaks near 260 and 240 nm, respectively, both in the absence and presence of porphyrin **5**, indicating parallel G-quadruplex (see Figure 2(a)). These spectral features were similar to those of d(T_2_G_4_)_4_. On the other hand, in the absence of added cation, CD spectrum of d(TG_4_T_2_G_4_T) was completely different from that of d(T_2_G_4_)_4_ without porphyrin **5**; while in the presence of **5**, the locations of positive and negative CD signals of d(TG_4_T_2_G_4_T) and d(T_2_G_4_)_4_ were almost identical (as in Figure 2(b)). These results indicate that, while these two sequences have different structure in water, both are induced to form parallel G-quadruplex by the addition of cationic porphyrin **5**.

In conclusion, the novel porphyrin carrying four cationic tethers **5** induced the formation of parallel G-quadruplex with *Tetrahymena* telomeric sequence. The effect of G-quadruplex induction was strong enough and made both sequences d(T_2_G_4_)_4_ and d(TG_4_T_2_G_4_T) folded into G-quadruplex without the addition of cation and buffer. The formation of the G-quadruplex under *in vitro* environment is mandatory for recent applications of G-quadruplex to nano-materials. Thus, the simple conditions for the formation of G-quadruplex are desired. The system described here must be useful for the development of nanobiomaterials. In more general, it will be important to control the structure of biomolecules with small organic compounds for nanobiotechnology.
